# Microneedle-Mediated Transdermal Delivery of Genetic Materials, Stem Cells, and Secretome: An Update and Progression

**DOI:** 10.3390/pharmaceutics15122767

**Published:** 2023-12-13

**Authors:** Avelia Devina Calista Nainggolan, Qonita Kurnia Anjani, Pietradewi Hartrianti, Ryan F. Donnelly, Arief Kurniawan, Delly Ramadon

**Affiliations:** 1 Faculty of Pharmacy, Universitas Indonesia, Depok 16424, Indonesia; avelia.devina11@ui.ac.id (A.D.C.N.); arief.kurniawan@farmasi.ui.ac.id (A.K.); 2 School of Pharmacy, Medical Biology Centre, Queen’s University Belfast, 97 Lisburn Road, Belfast BT9 7BL, UK; qonita.anjani@qub.ac.uk (Q.K.A.); r.donnelly@qub.ac.uk (R.F.D.); 3 School of Life Sciences, Indonesia International Institute of Life Sciences, Jakarta 13210, Indonesia; pietradewi.hartrianti@i3l.ac.id

**Keywords:** microneedles, biopharmaceutics, genetic materials, stem cell, secretome

## Abstract

Medical practitioners commonly use oral and parenteral dosage forms to administer drugs to patients. However, these forms have certain drawbacks, particularly concerning patients’ comfort and compliance. Transdermal drug delivery presents a promising solution to address these issues. Nevertheless, the *stratum corneum*, as the outermost skin layer, can impede drug permeation, especially for macromolecules, genetic materials, stem cells, and secretome. Microneedles, a dosage form for transdermal delivery, offer an alternative approach, particularly for biopharmaceutical products. In this review, the authors will examine the latest research on microneedle formulations designed to deliver genetic materials, stem cells, and their derivatives. Numerous studies have explored different types of microneedles and evaluated their ability to deliver these products using preclinical models. Some of these investigations have compared microneedles with conventional dosage forms, demonstrating their significant potential for advancing the development of biotherapeutics in the future.

## 1. Introduction

There are 33 cellular and gene therapy products that have been approved by the United States Food and Drug Administrator (FDA), with the majority of them being administered through parenteral injection [1]. The parenteral route has gained preference over common options, like oral administration. This is because peptides and proteins are susceptible to degradation in the gastrointestinal tract and undergo first-pass metabolism in the liver [2]. The parenteral route remains the gold standard for these compounds [3]. Additionally, intravenous injection within the parenteral route ensures optimal (100%) drug bioavailability, as there is no absorption phase after administration [4]. As a result, the parenteral route has emerged as the primary choice for delivering cellular and genetic-based medical treatments [5]. However, the parenteral does come with its limitations. These include pain at the application site, the need for medical personnel for application, and the generation of needle waste [4,6,7]. Furthermore, cellular and gene therapy products often require multiple doses due to their short half lives. This frequent dosing can lead to poor patient compliance, as patients may find the treatments uncomfortable [5]. Additionally, peptide compounds in the form of liquid injections or lyophilised dosage forms require a cold storage condition. This presents challenges in terms of manufacturing facilities and cold chain distribution, particularly in tropical countries, like Indonesia [8]. Consequently, alternative delivery routes are needed to address these issues.

The transdermal route offers a potential solution to the challenges mentioned above. This approach is less invasive and pain-free compared to the parenteral route [6,9]. Transdermal drug delivery involves using healthy skin as the application site, allowing drugs to enter the systemic circulation through diffusion or percutaneous absorption [10]. The skin, being the body’s largest organ, provides a variety of application areas and is easily accessible to patients [11]. Given the presence of blood vessels and lymphatics in the dermal layer, transdermal drug delivery can still achieve systemic effects [11]. This review encapsulates the latest research on the microneedle-mediated transdermal delivery of genetic materials, stem cells, and their products.

## 2. The Transdermal Route as an Alternative for Biomacromolecules Delivery

The skin comprises several layers: the epidermis, dermis, and subcutaneous [12]. The basal membrane separates the epidermis and dermis layers, while there is no membrane between the dermis and subcutaneous layer [11]. The epidermis, the outermost layer, consists of four stratified layers: the *stratum corneum* as the outermost layer, followed by the *stratum granulosum*, *stratum spinosum,* and the *stratum basale* as the bottom [12,13]. The dermis layer contains various skin appendages such as hair follicles, sebaceous glands, eccrine and apocrine sweat glands [11]. It also houses sensory nerve endings, lymphatic vessels, and blood vessels connected to systemic circulation [13]. The subcutaneous layer, known as the hypodermis, is the deepest layer and consists of adipose cells or fat [11,12].

**Figure 1:**
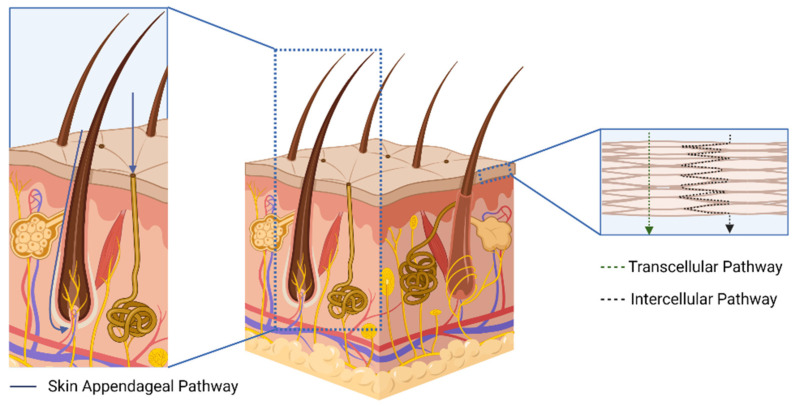
Schematic illustration of drug permeation on human skin.

Transdermal drug delivery can occur through two main pathways: diffusion through the epidermis layers (intercellular and transcellular) or via skin appendages, such as hair follicles and sweat glands, which is illustrated in Figure 1 [12]. In the intercellular pathway, drug molecules move through the gaps between corneocyte cells. In the transcellular pathway, drug molecules partition into corneocyte cells that are primarily composed of keratin. Additionally, drug molecules can take shortcut paths through hair follicles and sweat glands, known as the trans-appendageal route [14]. The choice of pathway depends on the patient’s skin condition and the physicochemical characteristics of the drug molecules [11]. The transcellular pathway is favorable for lipophilic drugs due to the composition of the *stratum corneum* membrane, while the intercellular pathway is better suited for hydrophilic drugs [14,15]. Despite the absence of the *stratum corneum*, the trans-appendageal pathway is not the primary route for drug permeation, as skin appendages only make up around 0.1% of the skin [11,12].

The outermost layer of the skin, the *stratum corneum*, acts as a barrier limiting drug penetration [11]. As a result, only a few molecules can pass through the *stratum corneum*, depending on their physicochemical characteristics [14]. Ideal drug candidates for transdermal delivery exhibit certain characteristics, including a molecular weight of less than 600 Da, proportional solubility in both oil and water with logP values between 1 and 3, and a preference for the unionized form of the drug [12,13,16]. Researchers are exploring advanced technologies to optimize the delivery of macromolecule drugs, including genetic material, stem cells, and their secretome products.

Researchers have devised various strategies to enhance the permeation of non-candidate skin drug molecules, utilizing both passive and active permeation methods. Passive methods involve formulating the drug with chemical penetration enhancers or inactive compounds that interact with specific components of the skin, particularly the lipid bilayer of the intercellular matrix in the *stratum corneum* membrane [9,16]. However, it is important to note that long-term modification of the skin’s normal state may disrupt the skin’s barrier function, potentially leading to irritation or toxicity effects on users [17]. Another strategy for enhancing drug permeation involves physical methods that utilize external resources, such as electrical, ultrasonic, or mechanical stimulations to temporarily disrupt the skin barrier [9,11,12,18]. Electric-mediated physical enhancer methods, like electroporation and iontophoresis, have shown the capability to enhance the permeation of both lipophilic and hydrophilic drug molecules in larger sizes [6,16]. Ultrasonic concepts are utilized in sonophoresis, where ultrasound energy is applied to the skin, raising the local temperature and subsequently enhancing permeability [18].

One of the recently developed promising strategies is microneedles, a dosage form comprising micron-sized needles (ranging from 100 to 1000 μm) on a baseplate that can be designed in various shapes [19]. Microneedles pierce the *stratum corneum*, specifically targeting the epidermis layer without reaching pain nerves and blood vessels [20]. Their shape allows for pain-free application and the creation of micropores, serving as new pathways for the penetration of hydrophilic and macromolecule drugs into the skin [12]. Microneedles offer a viable option for delivering a wide range of drug and biopharmaceutical molecules [21,22,23,24,25,26,27,28]. Importantly, patients do not require medical assistance to apply microneedles, improving patient compliance [29,30]. Effective insertion without bending or breaking relies on factors like needle height, tip radius, spacing, geometry, and density [20]. Larger and longer microneedles lead to enhanced skin permeability by reducing the drug’s diffusion path [14,31]. However, researchers need to consider insertion depth to avoid pain nerve and blood vessels [12,13]. Tip needle diameter less than 15 μm and larger spacing between needles result in easier penetration [32]. Needle shapes can be designed in various models such as conical, pyramidal, and bevel-tipped or arrowhead forms [19]. The pyramidal shape offers greater mechanical strength compared to the conical due to its larger cross-sectional area and aspect ratio between length and base diameter [32]. Increasing the number of needles in a dosage form enhances skin permeation, although excessive needles could require high insertion force leading to a “bed of nails” effect. Hence, flexibility is crucial to accommodate skin elasticity [19,20]. Microneedles design is more straightforward compared to other advanced transdermal technologies, like electrophoresis, iontophoresis, or ultrasound, which necessitate special instruments [17]. With the potential outlined above, the delivery of macromolecules using microneedles holds promise for development.

## 3. Types of Microneedles and the Use of the MN Platform to Deliver Genetic Materials, Stem Cells, and Secretome

**Figure 2:**
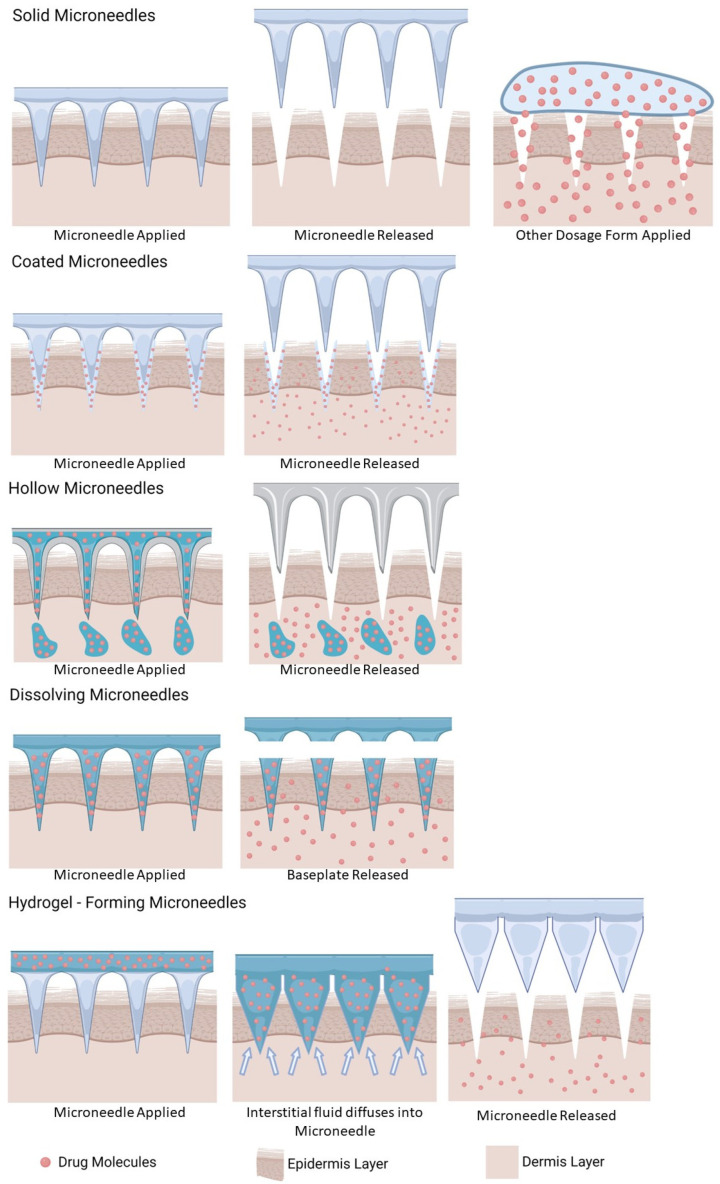
Schematic illustration of the types of microneedles.

There are five types of microneedles based on their delivery systems: solid, coated, hollow, dissolving, and hydrogel-forming microneedles, which are described in Table 1 and Figure 2. The solid microneedle system is relatively simple; the needles are typically made from silicon or metal, creating micropores in the skin. Patches or topical dosage forms loaded with drugs are placed over these micropores allowing drug diffusion into skin layers [14]. However, this approach involves a two-step application. To streamline this, the coated microneedle was developed [19]. In coated microneedles, drugs are coated onto the needle surfaces. As the microneedle is applied to the skin, the drug is deposited and dissolved in the skin after insertion [14]. Coated microneedles drug formulations need suitable viscosity to remain attached to the needle surface during storage and application on the skin. However, due to the small surface of the needle, the drug content that can be delivered is limited, making it unsuitable for drugs requiring higher doses [12,14,33]. Another type is the hollow microneedle, which features a drug chamber and a needle design similar to a syringe’s needle (with a hole in the middle). This allows the drug from the chamber to flow into the skin when the microneedles are applied. The drug chamber on the hollow microneedle’s feature can overcome the previous model’s limitation by increasing the drug holding capacity [14]. Dissolving microneedles are composed of both drugs and polymers in a single unit resulting in a decreased drug loading ability [9,14]. When applied to the skin, interstitial fluid dissolves the needle, releasing the drug from the dosage form into the skin [14,34]. Lastly, hydrogel-forming microneedles have a drug reservoir that conquers limited drug-holding capacity, with the separated needle’s part made from hydrogel polymers [14]. Upon application to the skin, the needles swell as interstitial fluid diffuses into the matrix. This swelling allows drug molecules from the reservoir to diffuse into the skin through swollen needles [35]. With respect to drug loading, needle geometry also has an impact on this aspect, regardless of the type of microneedle. Drug loading can also be improved by increasing the base width, height, and shape of the needle [36].

Several techniques to produce microneedles can be selected based on the incorporated materials. Laser cutting and dry or wet etching methods can be applied to solid, coated, or hollow microneedles, which are often made of metal or silicon materials [14]. However, there is an additional process for coated microneedles to attach the drug layer to the needle’s surfaces using spray or the repeated dip coating procedure [12]. Hollow microneedles can also be made from glass material which can be formed by dry or wet etching and micropipette puller technologies [14,33]. Meanwhile, polymer-based microneedle, coated, dissolving, and hydrogel-forming microneedles can be fabricated using solvent casting and lithography methods, which can be modified depending on the characteristics of the polymer [37,38,39,40].

**Table 1:** Summary of the materials, application, strength, and weaknesses of microneedle types.

Microneedle Type	Material	Application	Advantages	Drawbacks
Solid Microneedles	Silicon, silica glass, and metals, such as stainless steel and titanium	Two-step application	▪ High mechanical strength for efficient penetration ▪ Combined with other dosage forms such as gel, creams, or patch ▪ Could be formed in various shapes or sizes	▪ Non-biodegradable materials, promoting sharp waste after application ▪ Made from brittle materials that may fracture after insertion and leave residues on the skin ▪ Need two steps for “poke with patch” application
Coated Microneedles	Ceramics, silicon, silica glass, and metals, such as stainless steel and titanium	Single-step application	▪ Need one step for “coat and poke” application ▪ Does not have an additional drug reservoir	▪ Non-biodegradable materials, promoting sharp waste after application ▪ Drug capacity is limited ▪ Complicated fabrication to ensure coating formulation attached at the needle surface
Hollow Microneedles	Ceramics, glass, and metals, such as stainless steel and titanium	Single-step application	▪ Simple and fast application ▪ Could deliver fluid formulation with a high dose ▪ Needle shape is similar to a conventional needle injection	▪ Non-biodegradable materials, promoting sharp waste after application ▪ Expensive and complex fabrication method ▪ Risk of blockage in the needle during application
Dissolving Microneedles	Biodegradable and biocompatible polymers	Single-step application	▪ Made from biodegradable materials, which results in no sharp waste after application ▪ Fabricated with a simple and inexpensive method ▪ Suitable for controllable drug release purposes ▪ Simple and fast application	▪ Drug capacity is limited ▪ To obtain sufficient mechanical strength and fast dissolution, it will need a thorough selection of materials
Hydrogel-Forming Microneedles	Crosslinking biocompatible polymers	Single-step application	▪ Could deliver high numbers of drug ▪ Combined with other dosage forms, such as a hydrogel patch loaded on the drug reservoir ▪ Controllable drug delivery kinetic ▪ Needle will swell after application; no risk for reinsertion, which decreases the risk of infection transmission	▪ Requires a long application time ▪ Some polymers are non-biodegradable, which promotes sharp waste after application

[9,11,13,19,32,41,42].

## 4. Genetic Material-Based Therapy

Gene therapy involves the concept of manipulating genetic material within cells to produce beneficial proteins for therapeutic purposes, disease prevention, or addressing abnormal genes [43]. This strategy aims to rectify mutated genes in human somatic cells by avoiding their transcription and translation, replacing them with healthy counterparts or introducing new genes for therapeutic benefits [44]. When genes are introduced into target nuclei, these cells begin to express specific proteins essential for managing particular diseases [45]. However, the delivery of nucleic acids, like DNA and RNA, which serve as genetic materials in gene therapy, poses challenges due to their inherent instability and difficulty in reaching target cells [46,47]. The complex interplay between intracellular and extracellular environments further complicates gene delivery [48]. The anionic and hydrophilic nature of nucleic acid, characteristics that hinder their efficient delivery to cell nuclei, adds to the challenge [49]. Consequently, gene delivery necessitates a systematic approach to achieve effective therapeutic outcomes with minimal side effects [47]. In this context, vectors, carriers designed to encapsulate genetic materials, play a pivotal role in enhancing the efficiency and specificity of delivering these materials into target cells [46,50].

**Figure 3:**
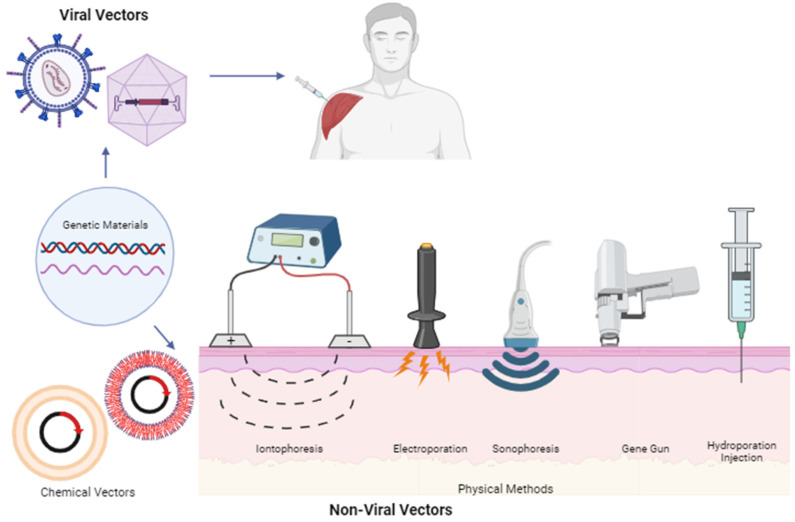
Illustration of genetic materials delivery methods.

Gene delivery involves two main methods: ex vivo and in vivo methods. In the ex vivo approach, vectors are directly introduced into target cells in a laboratory setting, which is summarized in Figure 3. The modified cells are then administered to the patient’s body. Conversely, in the in vivo method, vectors encapsulating the gene code are administered directly to the patients [46]. Selecting the appropriate vector is crucial in gene therapy design, as it dictates how genetic materials are delivered. Vectors are broadly categorized into viral and non-viral types [50]. Viral vectors utilize the inherent replication abilities of viruses to target and infect host cells, making them advantageous for gene delivery [45]. Examples of viral vectors include adenovirus, AAV (Adeno-Associated Virus), and Retrovirus [46]. Several commercial COVID-19 vaccines, such as the Janssen COVID-19 Vaccine, Oxford–AstraZeneca COVID-19 Vaccine, Sputnik V, Sputnik Light, and Convidecia, utilize adenovirus vectors to deliver the gene encoding the spike protein from SARS-CoV-2 [51]. Luxturna, a gene therapy for retinal dystrophy, employs AAV as a vector to deliver the RPE65 gene code, which replaces the mutated gene [52]. Retrovirus vectors, including Strimvelis^®^ (AGC Biologics S.p.A., Milan, Italy), are used to deliver the cDNA sequence of the adenosine deaminase enzyme to treat adenosine deaminase deficiency–severe combined immunodeficiency (ADA-SCID) [53].

In contrast to viral vectors, non-viral vectors are delivery systems that do not rely on viruses. This category is divided into physical methods and chemical methods. The physical method encompasses techniques such as needle delivery, ballistic DNA, electroporation, sonoporation, photoporation, magnetofection, or hydroporation. Chemical methods involve carriers made from lipids (lipoplex, liposomes, or solid lipid nanoparticles) or polymers (polyethyleneimine (PEI), chitosan, or poly(lactic-co-glycolic acid) (PLGA)) [45,54]. For example, naked DNA expressing IL-12 was delivered via intramuscular injection in a mouse model to prevent angiogenesis and suppress tumor growth [55]. The helium-driven gene gun delivery of ballistic pDNA containing the *Helicobacter pylori* outer inflammatory protein (oipA) along with IL-2 and LTB gene plasmid induced an immune response against *H. pylori* infection [56]. Electroporation devices like TriGrid^®^ (Ichor Medical Systems, Inc., San Diego, USA) enhanced antigen-specific HIV-1 production after intramuscular injection of HIV-1 pDNA in healthy human volunteers [57]. A combination of plasmids for granulocyte–macrophage colony-stimulating factor (pGM-CSF) and anti-programmed death 1 antibody (aPD-1) reduced tumor growth upon the application of ultrasound (sonication) following intratumoral injection in a breast cancer mouse model [58]. The intramuscular injection of miRNA-139-5p and anti-miR-139-5p followed by magnetic stimulation repaired rats’ internal anal sphincter (IAS) tone [59]. Minicircle naked DNA vectors containing Phenylalanine hydroxylase cDNA were successfully delivered to an inherited liver deficiency mouse model using hydrodynamic tail vein (HTV) injection [60]. Cationic lipid nanoparticles are used as vectors for Pfizer/BioNTech’s BNT162b2 vaccine and Moderna’s mRNA1273 vaccine, both delivering mRNA encoding the SARS-CoV-2 spike protein as COVID-19 vaccines [49]. Nanoparticles made from galactose-grafted PEG and low molecular weight PEI were developed as vectors to deliver the IL15 plasmid, effectively treating orthotopic hepatocellular carcinoma in a mouse model [61]. Where viral vectors exhibit higher delivery efficiency, they come with limitations, such as the patient’s immune reactions and cytotoxic effect [50]. The non-viral vectors listed above still require invasive methods and non-practical devices for effective delivery.

In the past few years, extensive research has been conducted on the delivery of genetic materials using microneedle devices. This research extends beyond cancer therapy and encompasses other diseases, like infection and regeneration therapy. Researchers have explored various types of microneedles, ranging from dissolving to coated microneedles. These investigations have been carried out through preclinical studies involving both healthy and diseased animal models. Some researchers have even compared microneedle delivery with traditional methods such as intramuscular, subcutaneous, or intravenous delivery. There is a possibility for microneedles to deliver multiple active compounds to achieve optimal efficacy. Table 2 provides an overview of these diverse studies on microneedle development, including different designs and material types.

## 5. Therapies Based on Stem Cells and Their Products

**Figure 4:**
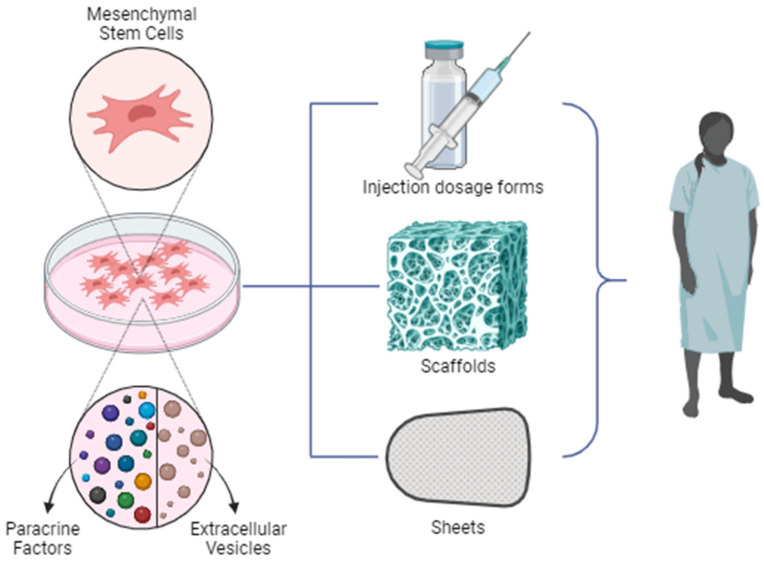
Illustration of mesenchymal stem cell (MSC) delivery methods.

Stem cells are non-specialized cells that can develop into specialized cells with specified functions [71]. They are known for their ability to replicate, regenerate, and differentiate into various types of cells within the human body as needed [72]. Therefore, stem cell-based therapy could serve as an alternative approach for diseases related to human genetic disorders, such as neurodegenerative diseases, heart disease, and osteoporosis [73]. Based on their potential, stem cells are categorized into four groups: totipotent, pluripotent, multipotent, and unipotent [74]. An example of totipotent stem cells is the zygote, resulting from the fertilization of an egg by a sperm, which can develop into various types of cells in an organism [74,75]. Pluripotent stem cells have the ability to differentiate into three germ layers, endoderm, mesoderm, and ectoderm, which form during the early stages of organ cell differentiation [74,75,76]. Hemopoietic stem cells fall into the multipotent stem cells category, as they can differentiate into specialized cells within a single lineage [74,75]. Unipotent cells, on the other hand, can differentiate into a single cell type, such as muscle stem cells [74,75]. Stem cells can be classified based on their sources, including embryonic stem cells, induced pluripotent stem cells, and adult stem cells [74]. Embryonic stem cells, an example of pluripotent stem cells, are found in the inner cell mass of a blastocyst, which is a developmental stage of the zygote before it implants in the uterus [76]. However, their use is restricted due to ethical concerns associated with obtaining them from embryo destruction [76,77]. Induced pluripotent stem cells are engineered stem cell created through gene modification to possess pluripotent capabilities, similar to embryonic stem cells [74,75]. Adult stem cells are present in the adult body and have the ability to self-replicate and differentiate into various cell types [74,75]. Among adult stem cells, hematopoietic stem cells can differentiate into all types of blood cells, while mesenchymal stem cells can differentiate into various germ layers [74,76]. Researchers often prefer mesenchymal stem cells due to their immunomodulatory properties, multi-differentiated capabilities, and support for the angiogenesis process [71]. Additionally, mesenchymal stem cells are readily available in various tissues, including bone marrow, skin, muscle, adipose tissue, and perinatal sources [78,79]. Despite their advantages, most FDA-approved stem cell-based products are administered via injection for local or systemic effect or they are used in scaffold applications, such as wound dressing or sheets, and placed in injured or target areas, as illustrated in Figure 4. These methods are still invasive and require complex procedures, including surgery [43]. Furthermore, there is an ongoing debate regarding the host’s immune responses to transplanted stem cells, as they may be recognized as foreign cells, potentially triggering immune reactions [76].

In addition to their established functions, stem cells have the capability to produce a substance known as secretome. Stem cells release this substance into the extracellular space, consisting of a group of bioactive factors that play roles in local or intercellular physiological processes [80,81]. Secretome contains a variety of compounds, including cytokines, growth factors, lipids, extracellular vesicles, and other metabolites, which are beneficial for promoting growth, proliferation, and differentiation within host cells [82]. One of the components of secretome, specifically paracrine factors, like growth factors, cytokines, hormones, and enzymes, can support mitogenesis and angiogenesis processes while preventing cell apoptosis [81,83]. These factors play crucial roles in the signaling process required in regenerative therapy, preventing cell apoptosis, stimulating cell proliferation, and facilitating the formation of blood to deliver nutrients to damaged tissues [71]. In addition to paracrine factors, extracellular vesicles are part of secretome and are divided into three subpopulations: exosomes, apoptotic bodies, and microvesicles. These extracellular vesicles serve as carriers for various growth factors such as FGF-2 (Fibroblast Growth Factor), HGF (Hepatocyte Growth Factor), and VEGF (Vascular Endothelial Growth Factor) [84]. Extracellular vesicles also play a role in removing unnecessary compounds from cells, which is essential for intercellular communication. Secretome-based regenerative therapy is considered more advantageous than using stem cells alone. Stem cells are known to be unstable during storage and transplantation and can pose risks of infection transmission and compatibility issues with the recipients’ immune system [82]. However, the composition of soluble factors and extracellular vesicles within secretome can vary based on their sources and environment. Therefore, achieving the desired components in secretome requires proper preparation and analysis [81].

The development of microneedles for the delivery of stem cells and their products has been relatively limited in the past five years. Moreover, the use of microneedles has been investigated as a method to administer non-cultured cell suspension for the treatment of vitiligo [85]. Nevertheless, in both preclinical and clinical examinations, certain studies have demonstrated comparable results between microneedles and other dosage forms, such as injection and topical. Additionally, there are opportunities to encapsulate stem cells or their products together with other drugs in a single dosage form, despite having similar therapeutic objectives, particularly in the field of regenerative therapy. These investigations have led to the development of various types of microneedles, including solid, dissolving, and hydrogel-forming microneedles. However, it is worth noting that some researchers employing solid microneedles have not provided comprehensive descriptions of the needle’s dimensions and materials, raising safety concerns, particularly regarding non-biodegradable material [15]. Table 3 presents several studies related to the development of microneedles for the delivery of stem cells and their products.

## 6. Future Considerations

This review underscores the numerous advantages of microneedles for delivering macromolecules via transdermal routes. However, it is essential for inventors, particularly during the development process, to take into account several drawbacks. Protein molecules, including genetic materials, stem cells, and their products, are susceptible to physical and chemical degradation throughout the stages of fabrication, distribution, and storage until application to patients. Such events can result in drug instability, reduced efficacy, or unforeseen adverse reactions [95]. Moreover, infection risks associated with microneedle application require safe sterilization methods for biopharmaceutical agents during development [96]. Additionally, transitioning from microfabrication to industrial-scale production poses complexity in the market. Some multiphase fabrication methods for microneedles are challenging to adapt to continuous-line manufacturing, leading to high production costs [19]. These manufacturing challenges and the associated high costs may deter pharmaceutical industries from investing in microneedle development, potentially impacting the affordability of these technologies for both patients and healthcare providers.

Despite these limitations, there is significant potential for genetic materials, stem cells, and their product therapies when incorporated into microneedles. Phase 1 clinical trials have been conducted to evaluate the safety and efficacy of the influenza vaccine, which was delivered by two types of microneedles: dissolving and coated microneedles [27,97]. Researchers and health regulators must consider various issues during the development process. Notably, microneedles, especially the dissolving and hydrogel-forming types, are susceptible to humid conditions, which can affect their mechanical strength [42]. Therefore, inventors should design appropriate packaging to ensure the reliability of dissolving and hydrogel-forming microneedles. Moreover, establishing minimum standards, such as quality control and aseptic protocols, is imperative to mitigate safety concerns related to the manufacturing process of microneedles [19].

## 7. Conclusions

Biopharmaceutical molecules, specifically genetic materials, stem cells, and their products, hold significant potential for development in disease prevention and therapy by addressing a wide range of conditions, including infection, cancer, and regenerative therapy. As previously mentioned, many cellular and gene therapy products approved by the US FDA are administered through parenteral dosage forms, which come with certain limitations, low patient acceptance rates, and the need for cold chain processes. Transdermal routes present attractive alternatives for delivering genetic materials, stem cells, and their products. However, the skin’s structure, includes the *stratum corneum*, the outermost layer, which acts as a barrier, impeding the entry of foreign molecules. Microneedles, serving as an innovative transdermal delivery system, can pierce the skin through the epidermis layer, creating bypass routes to facilitate the delivery of various types of drug molecules. Consequently, microneedles have the potential to incorporate macromolecules, such as genetic materials, stem cells, and their products, enabling transdermal delivery. Several studies focusing on the development of microneedles for the delivery of genetic materials, stem cells, and their products have been conducted in animal models. Various types of microneedles have been developed, including coated, dissolving, and hydrogel-forming microneedles. The results from these studies demonstrate similarities to conventional delivery methods, including parenteral and topical administration.

## Figures and Tables

**Table 2:** Various studies on the development of microneedles for genetic materials delivery.

Microneedle Design	Material	Compounds	Outcome	Ref.
5 × 5 microneedle arrays with each needle’s height being 1000 μm	Hyaluronic acid	p53 DNA and IR820	Dissolving microneedle patch containing p53 DNA and IR820 application with near-infrared laser irradiation could minimize tumor volumes in epidermoid carcinoma model mice compared to caudal vein injection treatment by promoting endosomal membrane obstruction and enhancing pDNA transfection efficiency.	[62]
19 × 19 microneedle arrays with each needle’s height being 600 μm	Poly(vinyl alcohol)	pDNA	A third immunization using a dissolving microneedle patch containing lyophilized RALA/pDNA nanoparticles could decrease tumor mass in cervical cancer model mice by increasing anti-E6/E7 IgG serum levels, which are higher levels than the intramuscular injection treatment group.	[41]
10 × 10 microneedle arrays with each needle’s height being 800 μm	Hyaluronic acid	miR-218	A dissolving microneedle patch containing miR-218 incorporated in lipid/polymer hybrid nanoparticles (LPNs) is applied every four days on shaved model mice compared to the gel formulation group, which results in fast-onset hair regrowth by promoting the proliferation of dermal papilla cells.	[63]
10 × 10 microneedle arrays with each needle’s height being 800 μm	Poly(vinylpyrrolidone)	Ovalbumin mRNA	After three immunizations by three dissolving microneedle patches carrying naked Ovalbumin (OVA) mRNA, tumor progression in E.G7-OVA carcinoma model mice have similar inhibition results with the subcutaneous injection method by increasing anti-OVA antibody production.	[64]
15 × 15 microneedle arrays with each needle’s height being 475 μm	Polypeptide copolymer matrix (mPEG_5K_-PN_2_LG_30_)	pOVA and poly(I:C)	Fourteen days after the fourth vaccination using a dissolving microneedle containing nanoplex pOVA and poly(I:C) loaded in a polypeptide copolymer matrix, B16-OVA melanoma model mice have an induced anti-OVA antibody IgG1 level higher than subcutaneous injection due to the contact of OVA antigens with antigen-presenting cells (APCs) located on dermal skin.	[65]
8 × 8 microneedle arrays with each needle’s height being 500 μm	Sodium hyaluronate	Ag85B DNA	Humoral immunities were formed on tuberculosis model mice which applied three dissolving microneedle patches containing Ag85B DNA before infection due to increasing IgG1 and IgG2a antibodies same level with intramuscular injection model mice.	[66]
12 × 12 microneedle arrays with each needle’s height being 650 μm	Dextran, poly(vinylpyrrolidone) and hyaluronic acid	STAT3 siRNA	A dissolving microneedle patch containing STAT3 siRNA encapsulated in polyethyleneimine (PEI) carrier could better result in reduced tumor volume and mass in B16F10 melanoma model mice than no treatment group by lowering STAT3 mRNA expression.	[67]
6 × 6 microneedle arrays with each needle’s height being 993 μm	Polycaprolactone, dimethulmaleic anhydride-modified polylysine (PLL-DMA), and polyethyleneimine (PEI)	p53 DNA	Oral carcinoma model mice have lower tumor growth rate after three applications of the coated microneedle patch containing p53 DNA with a stimulus-responsive transition layer (PLL-DMA) than the intravenous injection model due to highly expressed P53 protein, which could disrupt cancer cell proliferation.	[68]
10 × 10 microneedle arrays with each needle’s height being 600 μm	Maltodextrin, sucrose, and fish gelatin	HBsAg	Seven weeks after the third immunization of the hepatitis B vaccine by dissolving microneedle patch, 11-week-old female mice have robust humoral and cellular immune responses similar levels with intramuscular injection due to the induction of the hepatitis B surface antigen (HBsAg) to dendritic cells, which processes and lead it to T cells.	[69]
76 microneedle arrays with each needle’s height being 1000 μm	Sucrose, poly(vinyl alcohol), deoxycholic acid (DCA), and polyetherimide (PEI)	DNA	The third immunization using a dissolving microneedle patch consists of a DNA vaccine (ligation of antigens mH1 and mH3 with an internal ribosome-entry site (IRES)) encapsulated on a DCA-PEI nanomaterial has better protective immunity than an intramuscular injection in mice against influenza A H1N1 and H3N2 infection by introducing the DNA vaccine to Langerhans cells, which can trigger T cells and B cells.	[70]

**Table 3:** Various studies on the development of microneedles for stem cells and their product delivery.

Microneedle Design	Material	Compounds	Outcome	Ref.
11 × 11 microneedle arrays with each needle’s height being 600 μm	Poly(lactic-co-glycolic-acid) (PLGA), poly(vinylpyrrolidone) (PVP), and poly(vinyl alcohol) (PVA)	rHuKGF	Dissolving microneedles could deliver recombinant human keratinocyte growth factor (rHuKGF) by in vitro evaluation using Parafilm^®^ M (Bemis Company, Inc., Sheboygan Falls, WI, USA) exposed in a phosphate-buffered saline solution (PBS).	[86]
15 × 15 microneedle arrays with each needle’s height being 600 μm	Keratin, cysteine, hyaluronic acid, and poly(lactic-co-glycolic-acid) (PLGA)	Exosomes and UK5099	Exosomes derived from human bone marrow mesenchymal stem cells and UK5099 drugs loaded in PLGA nanoparticles incorporated on hydrogel microneedles have a higher hair regrowth effect than topical subcutaneous injection on hairless model mice after two rounds of application by activating hair follicle stem cells (HFSCs).	[87]
8 × 8 microneedle arrays with each needle’s height being 700 μm	Gelatin methacryloyl (GelMA) and poly(lactic-co-glycolic-acid) (PLGA)	MSCs	Detachable microneedles containing human bone marrow mesenchymal stem cells regenerate skin wound model mice by enhancing re-epithelialization and angiogenesis, which is compared to the intradermal injection model.	[88]
20 × 20 microneedle arrays with each needle’s height being 600 μm	Gelatin methacrylate (GelMA) and silk fibroin-methacryloyl (SilMA)	AgNPs and Exosomes	The single use of hydrogel microneedles containing exosomes derived from human umbilical cord mesenchymal stem cells and Ag nanoparticles has a faster wound healing process than injection at the wound site and antiinfection effect on wound-infected diabetic rats by improving the vascularization process and reducing inflammatory response.	[89]
15 × 15 microneedle arrays with each needle’s height being 600 μm	Hyaluronic acid, trehalose, and poly(vinylpyrrolidone) (PVP)	Secretome	After three applications of dissolving microneedles containing secretome from rat bone marrow mesenchymal stem cells, hairless model mice show higher hair regeneration effects than intradermal injection by enhancing angiogenesis around hair follicles.	[90]
15 × 15 microneedle arrays with each needle’s height being 600 μm	Hyaluronic acid	Extracellular vesicles	Dissolving microneedles could retain extracellular vesicles derived from human adipose stem cells longer in dermal fibroblasts in healthy mice than intradermal injection, which promotes collagen synthesis and fibroblast proliferation.	[26]
MN roller device	Not described	Extracellular vesicles	A combination solid microneedle roller with an extracellular vesicles solution derived from adipose stem cells topically applied on photoaging hairless model mice shows a better skin regeneration effect than the no treatment group by promoting the proliferation and migration of epidermal cells and fibroblasts.	[91]
Derma-Q^®^ (Dongbang Medi-Care Inc., Seongnam-si, Republic of Korea) device	Stainless steel	Secretome	Six topical administrations of secretome obtained from adipose stem cells after solid microneedle application on middle-aged Asian women provides anti-aging and whitening effects by increasing type I collagen expression and inhibiting melanin synthesis more than no treatment group.	[92]
36 microneedle arrays with each needle’s height being 150 μm	Not described	Secretome	Two weeks after facial treatment with topical concentrated secretome extracted from adipose stem cells assisted by solid microneedles reduced the wrinkle area in middle-aged Indonesian women due to extending procollagen type I production and had fewer side effects than those assisted by a fractional laser.	[93]
40 × 40 microneedle arrays with each needle’s height being 600 μm	Poly(vinyl alcohol) (PVA) and hyaluronic acid	Exosome and chitosan lactate	Two applications of dissolving microneedles containing calcium lactate and exosomes acquired from adipose stem cells results in better hair regeneration than the subcutaneous injection method on hair-shaved model mice by hair follicle stromal cell activation and modulation.	[94]

## Data Availability

Not applicable.
